# Effects of Conservation Tillage on Topsoil Microbial Metabolic Characteristics and Organic Carbon within Aggregates under a Rice (*Oryza sativa* L.) –Wheat (*Triticum aestivum* L.) Cropping System in Central China

**DOI:** 10.1371/journal.pone.0146145

**Published:** 2016-01-05

**Authors:** Li-Jin Guo, Shan Lin, Tian-Qi Liu, Cou-Gui Cao, Cheng-Fang Li

**Affiliations:** 1 MOA Key Laboratory of Crop Ecophysiology and Farming System in the Middle Reaches of the Yangtze River/College of Plant Science & Technology, Huazhong Agricultural University, Wuhan 430070, P.R. China; 2 Hubei Collaborative Innovation Center for Grain Industry, Yangtze University, Jingzhou 434023, Hubei, P.R. China; 3 College of Resources and Environment, Huazhong Agricultural University, Wuhan 430070, P.R. China; Chinese Academy of Sciences, CHINA

## Abstract

Investigating microbial metabolic characteristics and soil organic carbon (SOC) within aggregates and their relationships under conservation tillage may be useful in revealing the mechanism of SOC sequestration in conservation tillage systems. However, limited studies have been conducted to investigate the relationship between SOC and microbial metabolic characteristics within aggregate fractions under conservation tillage. We hypothesized that close relationships can exist between SOC and microbial metabolic characteristics within aggregates under conservation tillage. In this study, a field experiment was conducted from June 2011 to June 2013 following a split-plot design of a randomized complete block with tillage practices [conventional intensive tillage (CT) and no tillage (NT)] as main plots and straw returning methods [preceding crop residue returning (S, 2100−2500 kg C ha^−1^) and removal (NS, 0 kg C ha^-1^)] as subplots with three replications. The objective of this study was to reveal the effects of tillage practices and residue-returning methods on topsoil microbial metabolic characteristics and organic carbon (SOC) fractions within aggregates and their relationships under a rice–wheat cropping system in central China. Microbial metabolic characteristics investigated using the Biolog system was examined within two aggregate fractions (>0.25 and <0.25 mm). NT treatments significantly increased SOC concentration of bulk soil, >0.25 aggregate, and <0.25 mm aggregate in the 0−5 cm soil layer by 5.8%, 6.8% and 7.9% relative to CT treatments, respectively. S treatments had higher SOC concentration of bulk soil (12.9%), >0.25 mm aggregate (11.3%), and <0.25 mm aggregate (14.1%) than NS treatments. Compared with CT treatments, NT treatments increased MBC by 11.2%, 11.5%, and 20%, and dissolved organic carbon (DOC) concentration by 15.5%, 29.5%, and 14.1% of bulk soil, >0.25 mm aggregate, and <0.25 mm aggregate in the 0−5 cm soil layer, respectively. Compared with NS treatments, S treatments significantly increased MBC by 29.8%, 30.2%, and 24.1%, and DOC concentration by 23.2%, 25.0%, and 37.5% of bulk soil, >0.25 mm aggregate, and <0.25 mm aggregate in the 0−5 cm soil layer, respectively. Conservation tillage (NT and S) increased microbial metabolic activities and Shannon index in >0.25 and <0.25 mm aggregates in the 0−5 cm soil layer. Redundancy analysis showed that the SOC and its fractions (DOC and MBC) were closely correlated with microbial metabolic activities. Structural equation modelling showed that the increase in microbial metabolic activities directly improved SOC by promoting DOC in >0.25 mm aggregate in the upper (0−5 cm) soil layer under conservation tillage systems, as well as directly and indirectly by promoting DOC and MBC in <0.25 mm aggregate. Our results suggested that conservation tillage increased SOC in aggregates in the topsoil by improving microbial metabolic activities.

## Introduction

Anthropogenic carbon dioxide (CO_2_) emissions into the atmosphere have increased significantly by 39% from 6.3 Gt C in 1994 to 8.7 Gt C in 2009 [1]. Reducing CO_2_ concentration to mitigate global climate change by carbon (C) sequestration has been a promising method [2]. Considerable attention has been given to the dynamics of soil organic C (SOC) stocks and its function in long-term C accumulation and sequestration of atmospheric CO_2_ for mitigating climate change, maintaining crop productivity sustainability, and increasing soil fertility [3]. Reasonable management practices, such as no tillage (NT) and residue returning (S), facilitate SOC sequestration in croplands [2].

Agricultural SOC accumulation is influenced by numerous factors, such as tillage practices [4,5], soil aggregate size [4,6], and microbial functional diversity [7,8]. Tillage practices can affect the stability or composition of SOC [4,6], and thus affect SOC concentration and SOC density of the plough layer [4]. Conventional intensive tillage (CT) can decrease soil aggregate stability and accelerate soil organic matter oxidation [9], thereby threatening sustainable crop production [10]. Sustainable soil management can be achieved through conservation tillage practices, including NT and crop residue returning [11]. Conservation tillage significantly reduces soil physical disturbance [12], promotes soil aggregation, and improves soil microorganism dynamics because of more beneficial environmental conditions [13,14]. Therefore, investigating the effects of conservation tillage on SOC is necessary for further understanding soil sequestration.

Soil aggregates that control the dynamics of soil organic matter and nutrient cycling are structural units within the soil [15]. The aggregate hierarchy model shows that soil C accumulation in a given system may comprise a hierarchy of biological processes at the spatial dimension of soil physical structure [16,17]. Ettema and Wardle [18] reported that soil biota should be recognized at different spatial scales to understand their functions better in the ecosystem. Zhang et al. [4] also reported that previous studies mainly focused on the effects of microorganisms on the vertical and horizontal orientations of soil profiles and ignored the effects on the micro-spatial dimension of soil physical structure. Therefore, investigation of SOC driven by soil microbial community processes within soil aggregates will help elucidate the regulation of soil biota in soil C storage.

Soil microorganisms significantly affect the health of an agroecosystem through their functions in residue decomposition and nutrient cycling, as well as their associations with other organisms [19]. The activities and compositions of soil microbial community and their interactions with environmental factors affect SOC dynamics and crop productivity [19,20]. Direct mmeasurements of metabolic diversity of soil microbial communities are likely to provide more relevant information regarding soil functions compared with measurements of species diversity [20] because soil microorganisms generally present in resting or dormant stages, in which they are not functionally active [21]. Biolog system, a rapid community-level approach for assessing patterns of sole C source utilization, is used to study microbial community metabolic activities [22,23]. Several studies used the Biolog system to differentiate microbial communities from diverse habitats [22,23]. However, only a few these studies determined the relationship between soil microbial metabolic activities and SOC, especially within aggregates, in rice–wheat cropping systems.

Rice–wheat cropping systems possess important functions in food security in Asia by providing food grains for more than 20% of the population worldwide [24,25]. Central China comprises the main production region of rice and wheat in China. In this region, the planting area of the system occupies approximately 16% of the total planting area of both crops [26]. The effects of conservation tillage on rice–wheat cropping systems are well demonstrated [14,25]. However, limited attention has been given to the relationship between SOC and microbial metabolic characteristics within aggregate fractions under conservation tillage in the rice–wheat system. Thus, we hypothesized that (1) microbial metabolic activity is improved by conservation tillage at the small-scale in soil in the plow layer, and (2) the microbial metabolic activity is correlated to SOC within aggregates under conservation tillage. So, this study aimed to assess the effects of tillage practices (i.e., NT and CT) and straw returning methods (i.e., preceding crop residue removal and returning) on microbial metabolic characteristics and SOC within aggregates and their relationships under a rice–wheat cropping system in central China. To test our hypotheses, structural equation modelling (SEM) was used to detect potential associations among tillage systems (straw systems), microbial metabolic activities, organic C fractions, and SOC to elucidate the relationship better between soil microbial metabolic diversity and SOC within aggregates.

## Materials and Methods

### Ethic Statement

The experimental site was located at the Huazhong Agricultural University Research Farm of Huaqiao Town in Wuxue City, Hubei Province, China (E29° 51′N, 115° 33′E), which belongs to Extend Service Center of Agricultural Technonlogy of Wuxue Agricultural Bureau, Hubei Province. This study was performed in cooperation with Huazhong Agricultural University and Extend Service Center of Agricultural Technonlogy of Wuxue Agricultural Bureau, Hubei Province. The farm operations of this experiment were similar to local farmers’ operations and the field experiment did not involve endangered or protected species. This experiment was approved by College of Plant Science and Technology, Huazhong Agricultural University and Extend Service Center of Agricultural Technonlogy of Wuxue Agricultural Bureau, Hubei Province.

### Experimental site

This site has a humid mid-subtropical monsoon climate with an average annual temperature of 17.8°C and an annual precipitation of 1361 mm, with most of the rainfall occurring between April and August. The paddy soil of the site is a silty clay loam classified as Gleysol (FAO classification) [14]. The main soil properties (0−20 cm depth) are as follows: pH 4.79; organic C, 16.89 g kg^−1^; total nitrogen (N), 2.20 g kg^−1^; total phosphorus (P), 0.45 g kg^−1^; and bulk density, 1.21 g cm^−3^. The experimental site was cultivated with a cropping system of rice (Huanaghuazhan, *Oryza sativa* L.) and wheat (Zhengmai 9023, *Triticum aestivum* L.). Rice seedlings were thrown in June, and grains were harvested in October each year. Wheat was directly seeded in October, and grains were harvested in June the following year.

### Experimental design

Field treatment was initiated in June 2011 following a split-plot design of a randomized complete block and with tillage practices (CT and NT) as main plots and straw returning methods [preceding crop straw removal (NS) and return (S)] as subplots. The experiment comprised four treatments, namely, CTNS, CTS, NTNS and NTS. Each treatment had three replications. Each sub-plot has an area of 90 m^2^. For CTNS and NTNS treatments, preceding crop residues were removed and were not returned to the field. For CTS and NTS treatments, preceding crop residues were chopped to approximately 5−7 cm in length. The chopped residues were subsequently mulched in soil for NT and incorporated into soil for CT. CT treatment was moldboard ploughed twice to a 20 cm depth before throwing of rice seedlings and once before sowing of wheat. Soil disturbance was not conducted for NT treatments. The C/N ratios of wheat and rice residues used were about 46 and 71, respectively.

Weeds were controlled by spraying 36% glyphosate at 3 L ha^−1^. Rice seedlings were manually thrown at a density of 190,000 seedlings ha^−1^ in June, and grains were harvested in October each year. Wheat was directly seeded at 150 kg ha^−1^ in October of each year, and grains were harvested manually in June the following year. The fields were moist because conventional irrigation–drainage practices were followed. However, these fields were non-waterlogged through intermittent irrigation (irrigated every three days to five days) during the rice growing season, except during tillering and maturing stages. Irrigation was not provided during the wheat growing season.

Fertilizers were broadcasted during crop-growing season, with rice receiving 180 kg N ha^−1^, 90 kg P_2_O_5_ ha^−1^, and 180 kg K_2_O ha^−1^, and wheat receiving 144 kg N ha^−1^, 72 kg P_2_O_5_ ha^−1^, and 144 kg K_2_O ha^−1^. During rice-growing seasons, N fertilizer was applied in four splits: 50% N (as 46% urea) was used as basal N, 20% at the mid-tillering stage, 12% at the jointing stage, and 18% at the earring stage. During wheat-growing seasons, N fertilizer was applied in three splits: 50% N was used as basal N, 30% at the tillering stage, and 20% at the boosting stage. Both P (as single superphosphate, 16% P_2_O_5_) and K (as potassium chloride, 60% K_2_O) fertilizers were merely applied as basal fertilizers immediately after throwing rice seedlings or sowing wheat.

### Soil sampling and analysis

Soil cores from between rows in each plot were collected immediately after wheat harvest in June 2013 using a soil core sampler (inner diameter = 7 cm) at random. Samples consisted of eight composite soil cores sectioned into 0−5 cm, 5−15 cm and 15−20 cm depth increments. After sampling, visible plant residues and stones were removed, and large soil clods were gently broken by hand. Soils were then sieved through a 5-mm screen for uniformity. Aggregates were separated following the dry-sieving method described by Gartzia-Bengoetxea et al. [27]. A total of 100 g (<5 mm) of air-dried soil fragments were placed in a nest of sieves mounted on Retsch AS200 Control (Retsch Technology, Düsseldorf, Germany). Sieves were mechanically shaken (amplitude = 1.5 mm) for 2 min to separate soil into >0.25 and <0.25 mm aggregates. The SOC concentration of aggregate fractions was determined using a FlashEA 1112 elemental analyzer (Thermo Finnigan, Italy).

Soil microbial biomass C (MBC) was determined via fumigation–extraction method [28]. Both fumigated and non-fumigated soils were extracted with 0.5 M K_2_SO_4_ for 30 min, and organic C in the soil extract was measured through oxidation with potassium dichromate and titration with ferrous ammonium sulfate [29]. MBC was calculated as *E*_*C*_/*K*_*EC*_, where *E*_*C*_ is the difference of organic C extracted from the fumigated and non-fumigated soil, and *K*_*EC*_ is 0.38.

Dissolved organic C (DOC) was determined as described by Jiang et al. [30]. Fresh field soil (equivalent to a 10-g oven-dry weight, 1:2.5 ratio) was shaken with water at 250 r min^−1^ for 30 min at 25°C and then centrifuged at 4,500 rpm min^-1^ for 10 min. The supernatant was collected and filtered with a 45-μm membrane filter. The DOC of the filtrate was analyzed through oxidation with potassium dichromate and titration with ferrous ammonium sulfate [29].

### Biolog analysis of soil

The metabolic characteristics of soil microbial communities were measured using ECO Biolog system microplates (Biolog Inc. Hayward, CA), which were used to determine the C source utilization pattern. The 96-well ECO microplate comprised three replicate wells, each replicate comprised 31 C substrates and a control well without C substrates. The substrates were carbohydrates (*n* = 12), amino acids (*n* = 6), carboxylic acids (*n* = 5), polymers (*n* = 4), phenolic compounds (*n* = 2), and amines (*n* = 2). Fresh soil samples weighing 1 g each were shaken in 99 ml of sterile saline water (0.85% NaCl w/v) for 30 min. The mixtures were then diluted to a final dilution of 10^−3^ in the plates. The plates were incubated at 25°C in the dark, and absorbance readings at 590 nm were determined at 0 h and every 12 h thereafter up to 168 h by using a microplate reader (ELISA reaction plate reader). An average well color development (AWCD) was calculated to determine the rate of color development on Biolog plates for each plate at each reading [31]. Data at 96 h were used to calculate AWCD. Shannon index was calculated to evaluate microbial metabolic diversity. This index was calculated as follows: *H* = −∑[*p*_*i*_×ln(*p*_*i*_)], where *p*_*i*_ is the ratio of activities on each substrate to the sum of activities on all substrates [32].

### Statistical analysis

All data were analyzed with SAS 9.0 (SAS institute, 1990). The data sets were analyzed as a split-plot design with tillage treatment as the main factor and residue returning as sub-factor. Normality of the residuals was tested with the Shapiro-Wilk test and homogeneity of variances with Levene’s test. Two-way ANOVA was conducted to analyze the effects of tillage, straw returning and their interactions on microbial metabolic activity, SOC, DOC and MBC within aggregates. Duncan's multiple range tests were performed to examine whether the differences between the mean values were statistically significant at a significance level of 0.05. Redundancy analysis was performed using CANOCO software to explain the relationship between SOC and microbial metabolic diversity. Grace et al. [33] emphasized that SEM is an advanced and robust multivariate statistical method that allows hypothesis testing of complex path-relation networks. In the present study, SEM was used to evaluate whether soil microbial metabolic diversity has significant effects on SOC in response to the conversion of non-conservation tillage to conservation tillage. A *priori* model was constructed according to a literature review and our knowledge of how these predicators are related. Tillage systems (Tillage), straw systems (Straw), Shannon index, DOC, MBC, and SOC were the predicators in the initial model. In the model, microbial metabolic diversity was represented by Shannon index. The analysis was performed with AMOS 7.0 software [34] using the ‘robust’ maximum likelihood estimation procedures. Several tests were used to assess model fit, i.e. the χ^2^-test, comparative fit index (CFI), goodness-of-fit (GFI) and root square mean error of approximation (RMSEA).

## Results

### SOC fractions

Compared with CT treatments, NT treatments did not affect SOC concentration of bulk soil in the 5−20 cm soil layer, but significantly increased the SOC concentration of bulk soil in the 0−5 cm soil layer (Table 1). In comparison with NS treatments, S treatments had not significant effects on SOC concentration of bulk soil in the 5−20 cm soil layer, but significantly enhanced the SOC concentration of bulk soil in the 0−5 cm soil layer (Table 1). Therefore, this study only investigated the effects of conservation tillage on microbial metabolic characteristics and the relationships between the metabolic characteristics and SOC within aggregates in the 0−5 cm soil layer.

**Table 1:** Changes in SOC fractions within aggregates under different tillage and residue treatments.

Organic C	Soil fractions	CTNS	CTS	NTNS	NTS	T	S	T×S
SOC (0−5 cm soil layer)	Bulk soil	19.60±0.55 d	21.29±0.12 b	20.33±0.46 c	21.75±0.18 a	*	*	ns
(g kg^−1^)	>0.25 mm	19.70±0.10 c	21.30±0.10 b	20.43±0.06 c	23.37±0.06 a	**	**	**
	<0.25 mm	17.28±0.06 d	19.48±0.12 b	18.41±0.17 c	21.24±0.18 a	**	**	**
SOC (5−10 cm soil layer)	Bulk soil	17.84±0.56 a	18.10±0.20 a	17.87±0.87 a	18.31±0.17 a	ns	ns	ns
(g kg^−1^)	>0.25 mm	/	/	/	/			
	<0.25 mm	/	/	/	/			
SOC (10−20 cm soil layer)	Bulk soil	15.67±0.47 a	15.97±0.41a	15.53±0.41 a	15.50±0.20 a	ns	ns	ns
(g kg^−1^)	>0.25 mm	/	/	/	/			
	<0.25 mm	/	/	/	/			
MBC (0−5 cm soil layer)	Bulk soil	1846±5.84 d	2366±38.58 b	2024±11.40 c	2657±28.71 a	**	**	*
(mg kg^−1^)	>0.25 mm	1962±3.68 d	2538±27.09 b	2173±57.73 c	2844±22.90 a	**	**	ns
	<0.25 mm	1517±10.56 c	1820±14.42 b	1758±11.33 b	2245±33.86 a	*	**	**
DOC (0−5 cm soil layer)	Bulk soil	1.09±0.04 d	1.33±0.03 b	1.22±0.03 c	1.56±0.04 a	**	**	ns
(g kg^−1^)	>0.25 mm	1.05±0.05 d	1.43±0.03 b	1.34±0.01 c	1.86±0.01 a	**	**	*
	<0.25 mm	0.89±0.03 d	1.10±0.02 b	1.01±0.02 c	1.25±0.02 a	**	**	ns

Different letters in a line denote significant differences among treatments.

**, *P*<0.01

*, *P*<0.05

ns, not significant. CTNS, conventional intensive tillage with straw removal; CTS, conventional intensive tillage with straw returning; NTNS, no-tillage with straw removal; tillage; NTS, no-tillage with straw returning. T, tillage; S, straw; SOC, soil organic C; MBC, microbial biomass C; DOC, dissolved organic C; values are mean ± standard errors.

In the 0−5 cm soil layer, NT treatments significantly increased SOC concentration by 5.8%, 6.8%, and 7.9% of bulk soil, >0.25 mm aggregate, and <0.25 mm aggregate, respectively, compared with CT treatments (Table 1). NT treatments significantly increased MBC of bulk soil, >0.25 mm and <0.25 mm aggregates by 11.2%, 11.5% and 20.0%, respectively, compared with CT treatments. DOC concentrations of bulk soil, >0.25 mm aggregate, and <0.25 mm aggregate under NT treatments were 15.5%, 29.5%, and 14.1% higher than those under CT treatments, respectively. In comparison with NS treatments, S treatments significantly increased SOC concentrations of bulk soil by 12.8%, >0.25 mm aggregate by 11.3%, and <0.25 mm aggregate by 14.1%. In addition, MBC of bulk soil, >0.25 mm aggregate, and <0.25 mm aggregate under S treatments were 29.8%, 30.2%, and 24.1% higher than those of NS treatments, respectively. S treatments exhibited 25.0%, 37.5%, and 23.2% higher DOC concentrations of bulk soil, >0.25 mm aggregate, and <0.25 mm aggregate compared with NS treatments, respectively. In the 0−5 cm soil layer, there were significant interactions of tillage and straw returning on SOC concentration of >0.25 mm and <0.25 mm aggregates, MBC of bulk soil and <0.25 mm aggregate, and DOC concentration of >0.25 mm aggregate.

### Biolog substrate metabolic activities

Carbon (C) substrates tested using Biolog were categorized into six groups, namely, carbohydrates, amino acids, carboxylic acids, polymers, phenolic compounds, and amines (Table 2). Microbial metabolic activities varied among different treatments and aggregate fractions. Conservation tillage significantly increased microbial metabolic activities in both >0.25 and <0.25 mm aggregates. Compared with CT treatments, NT treatments significantly increased AWCD and Shannon index by 20.0% and 1.1% in >0.25 mm aggregate and by 23.0% and 1.3% in <0.25 mm aggregate, respectively. Similarly, compared with NS treatments, S treatments significantly increased AWCD and Shannon index by 37.5% and 2.4% in >0.25 mm aggregate and by 35.5% and 2.9% in <0.25 mm aggregate, respectively. In generally, interactions of tillage and straw returning significantly affected soil microbial metabolic activities and Shannon index in >0.25 mm and <0.25 mm aggregates in the 0−5 cm soil layer.

**Table 2:** Changes in microbial substrate utilization pattern and Shannon index within aggregates in the 0–5 cm soil layer under different tillage and residue treatments.

C sources	Soil fractions	CTNS	CTS	NTNS	NTS	T	S	T×S
Carbohydrates	>0.25 mm	8.07 ±0.07 d	10.71±0.11 b	9.31 ±0.08 c	12.68±0.11 a	**	**	**
	<0.25 mm	5.14 ±0.10 c	7.60 ±0.01 b	6.98 ±0.03 b	8.74 ±0.10 a	**	**	**
Amino acids	>0.25 mm	3.45 ±0.02 d	6.20 ±0.06 b	5.00 ±0.09 c	7.01± 0.10 a	**	**	**
	<0.25 mm	2.25 ±0.01 d	4.56 ±0.06 b	3.39 ±0.07 c	4.72±0.13 a	**	**	**
Carboxylic acids	>0.25 mm	3.32± 0.02 d	4.88 ±0.15 b	4.09 ±0.06 c	5.64 ±0.09 a	**	**	ns
	<0.25 mm	2.29 ±0.02 d	3.42 ±0.02 b	2.83 ±0.04 c	3.88 ±0.18 a	**	**	**
Polymers	>0.25 mm	4.22 ±0.03 c	5.23 ±0.04 b	4.92 ±0.03 b	6.55 ±0.07 a	**	**	*
	<0.25 mm	2.73 ±0.05 d	3.22 ±0.02 c	3.49 ±0.07 b	3.88 ±0.04 a	*	**	ns
Phenolic compounds	>0.25 mm	1.23±0.06 d	1.38±0.02 c	1.50±0.01 b	1.67±0.03 a	**	**	ns
	<0.25 mm	0.87± 0.05 c	0.84± 0.01 c	1.32±0.02 b	1.45±0.01 a	**	*	**
Amines	>0.25 mm	1.07±0.06 c	1.16±0.02 c	1.30±0.01 b	1.53±0.01 a	**	**	*
	<0.25 mm	0.41±0.01 c	0.60±0.02 b	0.58±0.01 b	0.69±0.01 a	**	**	**
AWCD	>0.25 mm	0.70±0.01 d	0.99±0.01 b	0.86±0.01 c	1.17±0.01 a	**	**	ns
	<0.25 mm	0.45±0.01 c	0.68±0.00 b	0.62±0.01 b	0.77±0.00 a	**	**	ns
Shannon index	>0.25 mm	3.18±0.00 c	3.26± 0.00 b	3.22± 0.00 b	3.29± 0.01 a	**	**	*
	<0.25 mm	3.07±0.01 c	3.18± 0.01 b	3.14± 0.00 b	3.20±0.00 a	**	**	**

Different letters in a line denote significant differences among treatments.

**, *P*<0.01

*, *P*<0.05

ns, not significant. CTNS, conventional intensive tillage with straw removal; CTS, conventional intensive tillage with straw returning; NTNS, no-tillage with straw removal; tillage; NTS, no-tillage with straw returning. T, tillage; S, straw; AWCD, an average well color development; values are mean ± standard errors.

**Fig 1:**
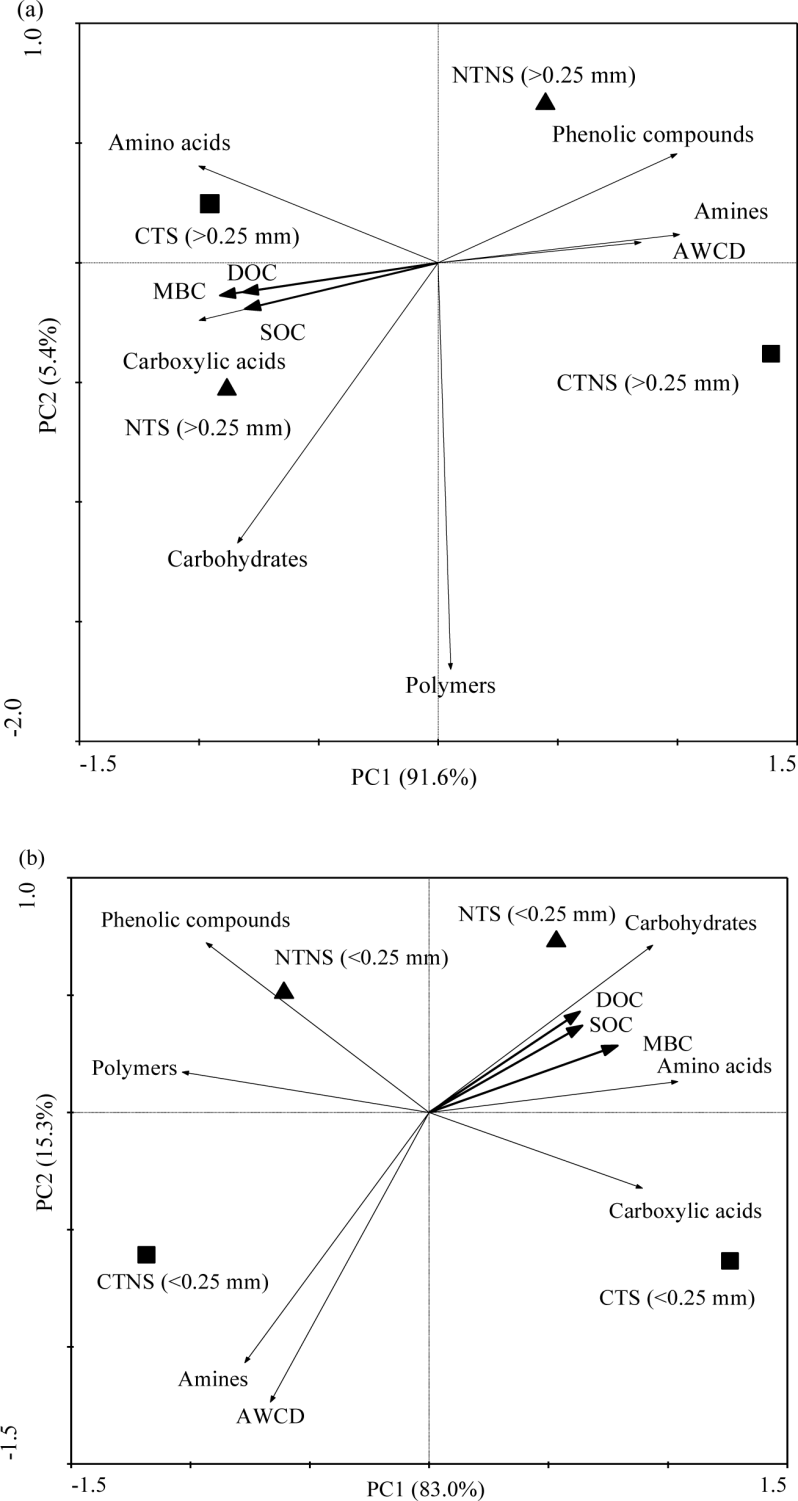
Redundancy analysis of soil microbial metabolic activities and SOC fractions in >0.25 mm (a) and <0.053 mm (b) aggregates under different tillage and residue treatments. CTNS, conventional intensive tillage with straw removal; CTS, conventional intensive tillage with straw returning; NTNS, no-tillage with straw removal; tillage; NTS, no-tillage with straw returning. SOC, soil organic C; MBC, microbial biomass C; DOC, dissolved organic C; AWCD, an average well color development.

Redundancy analysis shows that the coordinates from the first two ordination axes explained 97.0% (first axis, 91.6% and second axis, 5.4%) and 98.6% (first axis, 83.0% and second axis, 5.3%) of the variances in >0.25 mm (Fig 1A) and <0.053 mm aggregates (Fig 1B). Moreover, Monte Carlo permutation test showed that all SOC fractions (including SOC) significantly affected microbial substrate utilization pattern (*P*<0.05) in >0.25 and <0.25 mm aggregates. Overall, a clear separation was observed between treatments, and similar responses of SOC fractions (SOC, MBC, and DOC) within aggregates to microbial substrate utilization were found.

**Fig 2:**
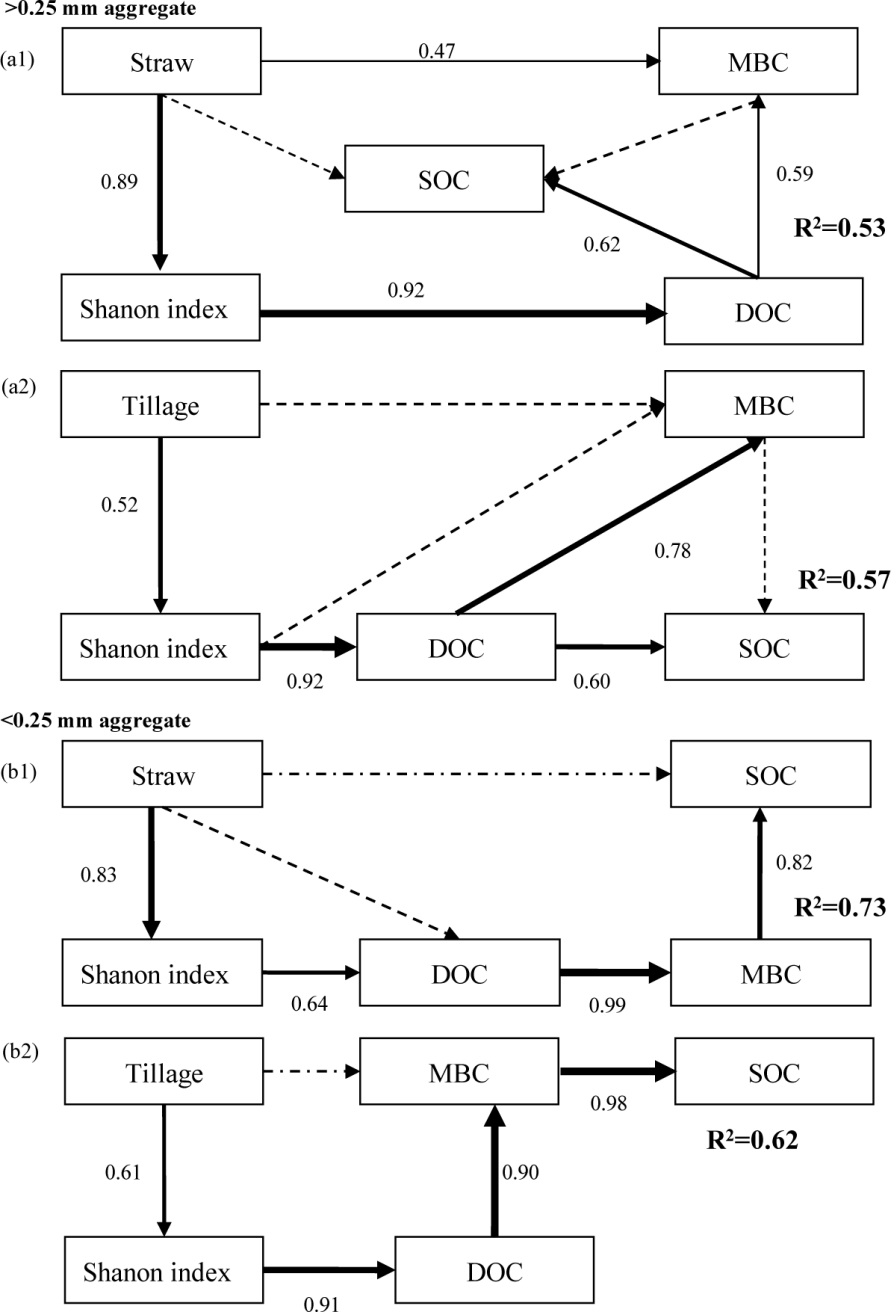
Structural equation modelling relating tillage systems, residue returning and microbial metabolic diversity to SOC in >0.25 mm (a1, χ^2^ = 2.791, df = 3, p = 0.432, CFI = 1, GFI = 0.920, RMSEA = 0.001; a2, χ^2^ = 6.163, df = 4, p = 0.617, CFI = 0.957, GFI = 0.978, RMSEA = 0.020) and <0.25 mm aggregates (b1, χ^2^ = 0.541, df = 4, p = 0.144, CFI = 0.956, GFI = 0.954, RMSEA = 0.010; b2, χ^2^ = 7.71, df = 4, p = 0.056, CFI = 0.975, GFI = 0.951, RMSEA = 0.020). Rectangles represent observed variables. Arrow thickness represents the magnitude of the path coefficient. Values associated with solid arrows represent the path coefficients. Solid and dashed arrows indicate significant (*P*<0.05) and non-significant (*P*>0.05), respectively. Tillage, tillage systems; Straw, straw systems; DOC, dissolved organic C; MBC, microbial biomass C; SOC, soil organic C.

Relationship between soil microbial metabolic diversity and SOC under tillage and residue systems were analyzed separately using SEM for >0.25 and <0.25 mm aggregates (Fig 2). SEM revealed that the predictors explained 53% to 57% of the variation in SOC in >0.25 mm aggregate, and 62% to 73% in <0.25 mm aggregate. In >0.25 and <0.25 mm aggregates, microbial metabolic diversity affected SOC directly and indirectly through DOC and MBC, respectively. Moreover, changes in microbial metabolic diversity induced by tillage or straw systems influenced SOC directly through DOC in >0.25 mm aggregate, and directly and indirectly through DOC and MBC in <0.25 mm aggregate (Fig 2).

## Discussion

This study investigated the effects of conservation tillage on soil microbial metabolic activities and SOC within aggregate fractions and their relationships under a rice-wheat cropping system in central China. The results partly supported our hypothesis that conservation tillage could only improve microbial metabolic activities and SOC in the top soil layer (0–5 cm) after two cycles of annual rice–wheat rotation and close relationships could be found between SOC and microbial metabolic activities within aggregates in this layer.

### Effects of conservation tillage on SOC, DOC, and MBC within aggregates

Conservation tillage generally increased SOC concentration of plow layer [10,35], which is probably because conservation tillage can reduce soil disturbance, promote root development in the topsoil, and increase crop residue accumulation on the soil surface, thus enhancing soil aggregate stability [9,10]. However, a number of studies reported no significant effects of conservation tillage on SOC [13,14]. Conservation tillage significantly increased SOC concentration of bulk soil in the 0−5 cm soil layer in this study (Table 1). This increase in SOC concentration can be attributed to a combination of less soil disturbance and more residues returned to the soil surface under conservation tillage [36,37]. Triberti et al. [38] reported that crop residues can significantly increase SOC concentration. Dikgwatlhe et al. [37] also reported similar results wherein conservation tillage increased SOC concentration in the 0−5 cm top soil. They suggested that the increase may be due to the lack of residues incorporated to soil and intensive soil tillage that accelerated soil organic matter decomposition. Alvarez et al. [39] found that NT increases SOC and total N concentrations in the first centimeters of the soil profile because NT maintains surface residues. However, similar results were not observed in the 5−20 cm soil layer (Table 1). The return of higher residues and root biomass to the soil surface instead of migrating into deeper soil under NT [36,40,41], weakening the effects of NT on the SOC content in the plow layer [37]. Although crop residues were incorporated into the plow layer in CTS treatment, soil disturbance would greatly decrease abundance and diversity of soil microorganisms [13,14], thus reducing decomposition of the residue incorporated into the deeper layer (5–20 cm). Therefore, no significant increases in SOC were found in the 5–20 cm soil layer among treatments.

In this study, SOC concentration was higher under >0.25 mm aggregate than that under <0.25 mm aggregate in the 0−5 cm soil layer (Table 1). Higher SOC concentrations generally are observed in macroaggregates than in microaggregates [42,43]. The conceptual model for aggregate hierarchy indicated that the increase in SOC concentration with increasing aggregate size is possibly caused by the macroaggregates, which are composed of the microaggregates plus organic binding agents [15]. The macroaggregates can provide better protection mechanism for soil organic matter than microaggregates [42,43]. Furthermore, higher SOC concentrations of >0.25 and <0.25 mm aggregates were observed under S treatments than under NS treatments, and under NT treatments than under CT treatments (Table 1). Madejon et al. [44] reported that soil biological activity under NT can promote the production of organic binding by-products that stabilize soil aggregates. Fresh and labile pools of organic matter cause rapid stimulation of soil microorganisms, accompanied by a significant increase in macroaggregate formation [45]. Zhao et al. [46] found that the majority of the increases in SOC under NT occur in macroaggregates. Moreover, NT and crop residue returning tend to reduce the turnover rate of macroaggregates [47], thus resulting in SOC accumulation in the soil surface. Jiang et al. [48] also reported that conservation tillage (NT and residue returning) promotes accumulation of macroaggregate-protected C.

In the current study, higher DOC and MBC of bulk soil, >0.25 mm aggregate, and <0.25 mm aggregate were observed under conservation tillage in the 0−5 cm soil layer (Table 1). Such increase may be attributed to higher organic matter inputs and improved environmental conditions for soil microbial community under conservation tillage [4].

### Effects of conservation tillage on soil microbial metabolic activities within aggregates

Nannipieri et al. [49] suggested that the activity and diversity of microorganisms determine the stability and function of soil ecosystems. Although the BIOLOG system has been criticized for being a cultivation-based method that considers only a fraction of all microbial species in soil [22,23], the system is widely used because it is an efficient method to reveal soil microbial C substrate metabolic activity [20,21]. Various studies have employed the microbial metabolic method to differentiate microbial communities among diverse habitats and examine the natural variation and diversity of microbial communities [23], which offers opportunities in monitoring changes in microbial diversity caused by different management practices [23]. Bending et al. [50] reported that soil management practices exhibit considerable influence on the structure and metabolic diversity of soil microorganisms. Garbeva et al. [51] found that residue quantity, quality, environmental conditions, and their complex interactions significantly affect soil microbial functional diversity.

Results of the present study showed that the Biolog system can reveal differences in microbial substrate utilization between treatments (Table 2). Conservation tillage significantly increased microbial substrate utilization (including amino acids, carboxylic acids, polymers, phenolic compounds, and carbohydrates) of >0.25 and <0.25 mm aggregates. This result suggests that conservation tillage can provide higher SOC availability from the decomposition of fresh organic inputs on the soil surface to soil microorganisms as suggested by Baker et al. [52]. Such findings are supported by high MBC and DOC under conservation tillage (Table 1). Moreover, conservation tillage improved soil microbial activity on the soil surface. Improvement in microbial activity leads to increased production of organic binding by-products from the decomposition of fresh organic residues that contributes to the presence of microaggregates and organic bindings that comprise macroaggregates [47,53]. Conservation tillage can also provide beneficial environmental conditions for soil microbial community through accumulating residues on the top soil layer and less fluctuation in soil water content and temperature [4,13]. Furthermore, conservation tillage can promote root development on the topsoil [54,55], thus providing high availability of organic C sources for soil microorganisms.

Shannon index reveals microbial species richness and evenness in terms of C component utilization [56]. Higher Shannon index was observed in >0.25 and <0.25 mm aggregates in the upper soil layer under conservation tillage (Table 2). Several researchers suggested that conservation tillage increases soil microbial abundance and diversity from various geographical locations, agroecosystem types, and cropping years [13,57]. For instance, Wang et al. [57] found higher soil bacterial abundance and diversity under NT and S treatments than under CT. They also reported that NT plus 100% crop residue incorporation is the best agricultural strategy for improving soil microbial communities, which may be due to the decrease in soil temperature and water content fluctuation, as well as increase in organic matter and energy sources provided by NT and residue incorporation. Lupwayi et al. [58] observed that catabolic diversity of microbial communities is greater under NT than under CT through the Biolog system. However, Helgason et al. [13] found that the distribution pattern of soil biota within aggregates may be governed by aggregate size and not by tillage practices. The discrepancy of the results may be attributed to soil-specific properties, such as mineralogy, soil C and nutrient concentration, and differences in the methods used [59,60].

### Relationship between SOC and microbial metabolic activities within aggregates under conservation tillage

Soil microbial communities possess important functions in SOC decomposition and C sequestration processes through metabolizing organic matter sources [19]. SOC is highly correlated with soil microbial community [4]. In this study, SOC was closely correlated with microbial substrate utilization (Fig 1). On one hand, SOC decomposition is controlled by the quality and availability of organic C resources utilized by microbial communities [13,19]. Several studies suggested that labile fractions (including MBC and DOC) are closely related to SOC dynamics [13,14]. On the other hand, soil microbial community and their interactions with the environment are important factors that affect SOC dynamics, and any change in soil microbial community may alter SOC availability [19].

A number of studies have reported that the distribution pattern of soil biota within aggregates may be governed by aggregate size [13]. Aggregate size is closely correlated with pore space, which determines the fluxes of oxygen and water [4,15], thus affecting soil biota and their functions in C dynamics. Stewart et al. [61] reported that soil C sequestration capacity is mainly determined by the degree of SOC protection from decomposition provided by the spatially hierarchical organization of soil aggregate structure. In the current study, metabolic diversity affected SOC directly and indirectly through DOC and MBC of >0.25 and <0.25 mm aggregates in the topsoil (0−5 cm) (Fig 2). Moreover, microbial metabolic diversity influenced SOC directly through DOC in >0.25 mm aggregate, and directly and indirectly through DOC and MBC in <0.25 mm aggregate under tillage and straw systems. Soil microenvironment contributes to the heterogeneous distribution of microorganisms within aggregates [62], thus leading to different effects of microorganisms on SOC within aggregates. Macroaggregates exhibit faster turnover time than microaggregates [63] because macroaggregates are mainly formed through binding of microaggregates and organic amendments [64]; therefore, macroaggregates more easily obtain fresh organic matter. Choudhury et al. [64] also reported that straw returning results in the preponderance of macroaggregates compared with microaggregates caused by the formation of water-stable aggregates. The presence of more stable macroaggregates is the first condition required for C sequestration [65]. Therefore, conservation tillage can be speculated to promote the accumulation of straws in the top soil layer (0−5 cm), which leads to rapid straw decomposition accompanied by microbial growth [66]. This phenomenon improves soil microbial metabolic activities. Straw decomposition also increases the input of labile organic matter (such as DOC and particulate organic C) into the soil and promotes the formation of >0.25 mm aggregate [67], eventually affecting SOC.

Compared with macroaggregates, microaggregates exhibit lower C concentrations and longer turnover times [68]. Aggregate size controls pore space of the aggregate, thus determining the fluxes of oxygen, water, and microhabitats for microbial biota [4,52]. Fungi reside in large pores of large macroaggregates because of relatively high aeration and SOC substrates [69,70]. By contrast, bacteria survive in microaggregates because microaggregates provide protective habitats for microorganisms through pore size exclusion of predators [71]. Zhang et al. [4] reported microbial contribution to SOC within aggregates. They found that bacteria and fungi contributed to SOC in >1 mm aggregate, whereas only bacteria were relevant in <1 mm aggregate. Zhang et al. [4] reported that soil microbial communities promote the accumulation of C directly and indirectly through MBC, and the input level of microbial-derived C and MBC regulate SOC within aggregates. In the present study, microbial functional diversity affected SOC directly and indirectly through DOC and MBC in <0.25 mm aggregate under tillage and straw systems (Fig 2). Therefore, conservation tillage can be speculated to increase organic matter input, thus promoting bacterial growth in <0.25 mm aggregate and subsequently SOC.

Several studies have reported that conservation tillage can decrease soil bulk density and improve soil porosity, thus promoting root development in the topsoil [54,55]. The root system is a main source of organic C accumulation in the soil [72], which directly influences the soil aggregation state. Uren [73] suggested that microbial function is largely affected by the ability of microorganisms to degrade root exudates, which are composed of small organic molecules, such as carbonic acids, amino acids, and sugars. Different tillage and straw managements can cause differences in physical aeration of soil conditions and in the growth of roots and their subsequent distribution [52]. This phenomenon leads to differences in plant root exudates within aggregate fractions and subsequently SOC. Baker et al. [52] also suggested that tillage practices can change soil environmental conditions, which affect root growth and C and N mineralization. Soil microbial community can respond quickly to the change [74], thus leading to shifts in soil microbial community components and microbial metabolic activities.

## Conclusions

After two cycles of annual rice–wheat rotation, results of the present study indicate that conservation tillage did not affect SOC in the plow layer (0−20 cm) but improved microbial metabolic activities and SOC in the upper (0−5 cm) soil profile. Close relationships between microbial metabolic activities and SOC within aggregates in the top soil layer. Results of SEM revealed that conservation tillage improved microbial metabolic diversity in the upper soil layer, thus increasing SOC of aggregates.

## Supporting Information

S1 File Original data of the manuscript. (XLSX)
